# Comparison of ZnO nanowires grown on e-beam evaporated Ag and ZnO seed layers†

**DOI:** 10.1039/c9na00553f

**Published:** 2020-05-19

**Authors:** Yulin Geng, Karina Jeronimo, Muhammad Ammar Bin Che Mahzan, Peter Lomax, Enrico Mastropaolo, Rebecca Cheung

**Affiliations:** Institute for Integrated Micro and Nano Systems, School of Engineering, University of Edinburgh, Scottish Microelectronics Centre Alexander Crum Brown Road Edinburgh EH9 3FF Scotland UK yulin.geng@ed.ac.uk

## Abstract

In this study, ZnO nanowires with diameters ranging from 50 nm to 500 nm have been synthesized hydrothermally on Ag and ZnO seed layers deposited by electron beam evaporation. ZnO nanowires grown on hetero and homo interfaces have been studied by comparing the growth characteristics of (a) ZnO nanowires on the Ag seed layer and (b) ZnO nanowires grown on the ZnO seed layer, respectively. The surface morphology of the as-evaporated seed layers before the nanowire growth has been investigated. Electron backscatter diffraction (EBSD) has been employed to examine the crystallinity of ZnO nanowires. In addition, the integrity of the Ag–ZnO heterointerface has been investigated using high-resolution transmission electron microscopy (HR-TEM). The length, diameter, density, and alignment of nanowires grown on Ag and ZnO seed layers have been studied as a function of growth time from 0.5 hours to 18 hours and precursor concentration from 5 mM to 18 mM. Furthermore, for both the Ag–ZnO nanowire heterostructure and ZnO–ZnO nanowire homostructure, the role of defects in the optical properties in the wavelength range of 517 nm to 900 nm has been studied using photoluminescence (PL) spectroscopy.

## Abstract 2

A systematic comparison between ZnO nanowires' hetero and homo growth: from the viewpoints of the growth mechanism, crystal quality, and optical properties.

## Graphical abstract

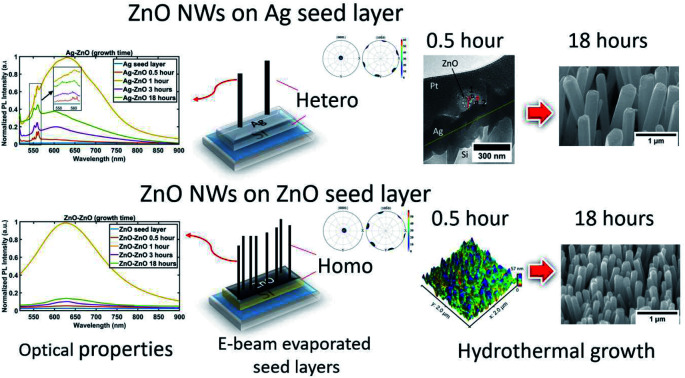

## I. Introduction

Recently, one dimensional (1D) materials with different properties have been employed to perform a range of functions. 1D Zinc Oxide (ZnO), as a wide band gap semiconductor with a 60 meV binding energy at room temperature,^1^ has been considered one of the most promising materials and has been developed for various applications such as piezoelectric,^2,3^ optoelectronic,^4,5^ and electrochemical devices.^6^ In the past decade, the hydrothermal growth, a low temperature bottom-up wet chemical synthesis method, of 1D ZnO nanostructures has drawn much attention. ZnO nanowires (NWs) have been grown hydrothermally on different substrates.^7–9^ Generally, a seed layer is required to grow NWs. In the literature, seed layers can be deposited by sol–gel^10^ (spin-casting the ZnO nanoparticles), sputtering,^11^ and vacuum evaporation^12^ techniques. The seed layers can be classified into two types: homo and hetero seed layers. For instance, ZnO nanoparticles^10,13^ or thin films^11^ can be treated as homostructures with ZnO NWs, while thin-film silver (Ag),^14^ gold (Au),^15^ copper (Cu)^16^ and gallium nitride (GaN)^17^ can serve as the heteroepitaxial seed layer to grow ZnO NWs due to the low lattice mismatch with the wurtzite crystal structure of ZnO. The hetero growth cases have also been called seedless growth.^17–19^ During the fabrication process, a proper seed layer should be chosen to meet the specific requirement for different applications. For example, ZnO NWs grown on a ZnO homo seed layer have been chosen as a photocatalyst due to the possibility of controlling the NWs' structures and the presence of fewer impurities associated with the ZnO NWs grown on the ZnO seed layer.^20^ However, the semiconducting characteristics of the ZnO thin-film seed layer may not be desirable under certain circumstances when direct electrical contact with low resistivity between ZnO NWs and the substrate is needed, such as in nano-generators and piezo-gated transistors.^18^ In such cases, metal hetero seed layers such as Ag thin films would be a better choice. Most recently, heterostructures based on ZnO NWs and other promising materials such as two-dimensional (2D) materials have drawn much interest due to the large potential in developing these material structures for different types of applications.^21–23^ However, study of the hydrothermal heteroepitaxy mechanism still requires more effort.

Over the past few decades, the sol–gel method and sputtering have been the most common methods to synthesize the thin-film seed layer for ZnO NW growth. The hydrothermal growth behaviour of ZnO NWs on different seed layers prepared with the sol–gel method and sputtering has been studied systematically by other researchers.^24,25^ The deposition parameters of the seed layer such as deposition rate can be adjusted to control the surface morphology, and subsequently, control the nature of the ZnO NWs. In our work, we introduce electron beam (e-beam) evaporated Ag and ZnO thin films as the seed layers to synthesize ZnO NWs. There are several reasons to use e-beam evaporation instead of the sol–gel method and sputtering as the deposition method. The sol–gel method can produce a ZnO homo seed layer only, and the use of alkaline solutions like sodium hydroxide (NaOH)^10^ or ammonium hydroxide^26^ to produce the ZnO nanoparticles may be incompatible with certain microfabrication processes. In addition, compared to sputtering, e-beam evaporation has a better directionality, and it is easier to control the crystalline structure and the surface roughness of the thin-film.^27^ As a very popular deposition method in current very-large-scale (VLS) microfabrication,^28^ e-beam evaporation can be an alternative method to prepare high-quality seed layers to grow ZnO NWs, and it is suitable for pattern definition in high-resolution lithography.^29,30^ However, studies on e-beam evaporated thin-films as the seed layer to grow ZnO NWs and the mechanism of ZnO NW growth on e-beam evaporated homo and hetero seed layers are still limited in the existing literature.

In this paper, ZnO NW growth on Ag and ZnO seed layers prepared by e-beam evaporation has been studied systematically. The surface roughness and grain size of the e-beam evaporated Ag and ZnO thin-film seed layers before NW growth have been investigated. The dependence of the initial nucleation of ZnO NWs on the surface properties of both Ag and ZnO seed layers has been investigated by studying the NWs grown within a 0.5 hour growth time. The Electron Backscatter Diffraction (EBSD) technique has been employed to examine the crystallinity of ZnO NWs grown on both Ag and ZnO seed layers. A high-resolution transmission electron microscope (HR-TEM) has been used to study the initial heterointerface between ZnO NWs and the Ag seed layer. The length, diameter, density, and alignment of nanowires on different seed layers as a function of growth time from 0.5 hours to 18 hours and precursor concentrations from 5 mM to 18 mM have been analysed. Furthermore, for both the Ag–ZnO NW heterostructure and ZnO–ZnO NW homostructure, the role of defects in the optical properties as a function of growth time and precursor concentration has been studied using photoluminescence (PL) spectroscopy in the wavelength range of 517 nm to 900 nm.

## II. Experimental

### A. Substrate preparation and e-beam evaporation of the Ag and ZnO seed layers

Firstly, 3 inch silicon (Si) wafers have been cleaned with acetone, isopropyl alcohol (IPA) and deionized (DI) water in an ultrasonic bay each for 3 min and dried with a N_2_ gun. A 10 nm titanium layer has been deposited to increase the adhesion between seed layers and Si substrates. Two different seed layers (Ag and ZnO) have been evaporated on two separate groups of samples. The starting vacuum pressure has been set at 2 × 10^−6^ torr, and the e-beam voltage has been fixed at 10 kV with ∼3% output power, which can result in a deposition rate of ∼1 Å s^−1^. The thickness of the seed layer thin-film has been controlled by deposition time. Ag and ZnO thin-films of 50 nm have been deposited. In addition, for the Ag hetero seed layer, samples with thicknesses from 50 nm to 100 nm have been used to investigate the relationship between the surface properties and the thickness of the Ag seed layer.

### B. ZnO NW hydrothermal growth

After the seed layer deposition, 3 inch Si wafers have been diced into 1 cm^2^ chips for hydrothermal growth. ZnO NWs have been grown hydrothermally in 1 : 1 zinc nitrate hexahydrate (Zn(NO_3_)_2_·6H_2_O) and HMTA (hexamethylenetetramine) mixed in DI water at 90 °C. Two groups of experiments have been performed on both Ag and ZnO seed layers as a function of (a) growth time (no preheating), which has been varied from 0.5 hours to 18 hours with the precursor concentration fixed at 40 mM; (b) the equimolar concentrations of the chemical precursors, which have been modified from 5 mM to 80 mM, with a fixed growth time of 18 hours.

### C. Characterisation

Atomic force microscopy (AFM) has been used to characterise the roughness and grain size of the as-deposited Ag and ZnO surface. After the hydrothermal growth, scanning electron microscopy (SEM), together with energy-dispersive X-ray spectroscopy (EDX), has been used to characterise the length, diameter, density, and alignment of the ZnO NWs. The data from SEM micrographs have been analysed using the ImageJ program. EBSD and cross-sectional HR-TEM have been used to examine the crystallinity of ZnO NWs. EBSD data have been analysed using the AZtecHKL program and MTEX program.^31^ For HR-TEM sample preparation, the focused ion beam (FIB) technique has been used to extract the ZnO NW–Ag heterointerface and transfer it to a copper grid; the details have been presented in the ESI.† In addition, PL spectra in the wavelength range of 517 nm to 900 nm have been obtained on both Ag seeded ZnO NWs and ZnO seeded ZnO NWs grown with different growth times and precursor concentrations.

## III. Results and discussion

### A. Surface roughness and grain size of the e-beam evaporated Ag and ZnO seed layer

The surface roughness and grain size of the e-beam evaporated thin-film seed layers before the hydrothermal growth of ZnO NWs have been investigated. Table 1 shows the average roughness and grain size of the Ag seed layer with thicknesses from 50 nm to 100 nm, together with a 50 nm ZnO seed layer. It can be seen that both 50 nm Ag and 50 nm ZnO thin-films are relatively smooth with a surface roughness between 1 nm and 2 nm. The roughness of all the seed layers deposited by e-beam evaporation is around 1 nm to 3 nm, and the grain diameter of all the seed layers is in the range between 28 nm and 44 nm. The observation of the similar surface roughness and grain size of all these seed layers is possibly because the evaporation rates for all the seed layers have been fixed at 1 Å s^−1^, consistent with the literature; the deposition rate is one of the most critical factors controlling the surface roughness and grain size of the e-beam evaporated thin-film.^32,33^

**Table 1:** Surface properties of evaporated seed layers and initial diameters of ZnO NWs^a^

The thickness of the evaporated seed layer	Average roughness *R*_a_ (nm)	Grain diameter (nm) (±10 nm)	0.5 hours of NW growth, average diameters of NWs (±10 nm)
50 nm Ag	1.19	30	130
75 nm Ag	1.52	28	170
100 nm Ag	2.60	44	260
50 nm ZnO	1.96	44	45

a The NWs' growth, with a precursor concentration of 40 mM, at 90 °C.

As for the influence of the thickness of the Ag seed layer on the surface roughness, the average roughness has been found to increase from 1.19 nm to 2.6 nm as the thickness of the Ag seed layer increases from 50 nm to 100 nm. In addition, no apparent relationship between the grain size and the thickness of the Ag seed layer has been found.

### B. The influence of the surface morphology of Ag and ZnO seed layers on the initial diameter of the as-grown ZnO NWs

**Fig. 1:**
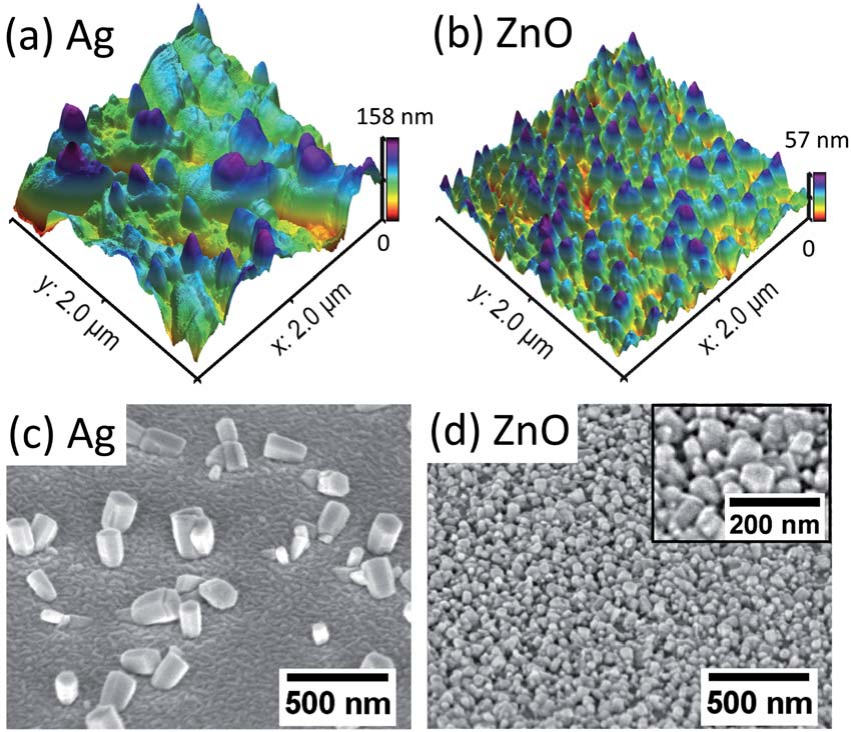
AFM and SEM (top view) images of the initial state (40 mM, 90 °C, 0.5 hours) ZnO nanocrystals grown on the 50 nm patterned Ag seed layer (a) and (c); ZnO seed layer (b) and (d). In (d), the high magnification SEM image is shown in the inset.

The dependence of the initial nucleation diameter of ZnO NWs on the surface morphology of Ag and ZnO seed layers has been studied by investigating the diameter of ZnO NWs with a 0.5 hour growth time. The reason for using the data of the 0.5 hour ZnO NWs' diameter is because 0.5 hours can be assumed to be the initial state of hydrothermal growth since it normally takes 6–18 hours to reach the saturation state of ZnO NW growth (a precursor concentration of 20 mM–40 mM and temperature of 90 °C).^24,34^Table 1 shows average diameters of ZnO NWs grown on Ag and ZnO seed layers after 0.5 hours. It can be seen in Table 1 that in general, for the Ag seed layers, the ZnO NWs have an average initial diameter of around 130 nm to 260 nm, which are about 4–6 times higher than the grain size of the Ag seed layer, while the ZnO NWs grown on the ZnO seed layer have a similar initial diameter to the grain size of the seed layer, which is around 45 nm. In addition, Fig. 1 shows the AFM and SEM images of NWs grown for 0.5 hours. It can be seen in Fig. 1 that the density of the NWs grown on the ZnO seed layer is significantly higher than that of NWs grown on the Ag seed layer. Meanwhile, NWs grown on the ZnO seed layer are observed to be more vertically aligned than NWs grown on the Ag seed layer. These observations are significant since the interaction of the interface between the ZnO NWs and the Ag (hetero) or ZnO (homo) seed layers appears to play an essential role in determining the initial diameter of ZnO NWs.

For ZnO NWs grown on the Ag seed layer, a heterointerface of Ag–ZnO would be required to be formed first during the initial state of hydrothermal growth; a ∼130 nm initial nucleation diameter of ZnO NWs has been observed on the Ag thin-film with a grain size of 30 nm, and the density of nucleated grains is observed to be much lower than that of ZnO NWs grown on the ZnO homo seed layer. The presence of lattice mismatch between the Ag thin-film and ZnO NWs will lead to a relatively large nucleation heterogeneous energy barrier,^35,36^ and the Ag grains have relatively random orientations. Therefore, compared to ZnO seeded NWs, a larger initial diameter may be formed to overcome the heterogeneous energy barrier and compensate for the randomly oriented Ag grains. Apart from ZnO NWs grown on the Ag hetero seed layer, in the literature, NWs grown directly on Si and graphene have also been reported to have a relatively larger diameter and lower density than homo seed growth under the same growth conditions.^35,37^ In addition, from our results in Table 1, the initial nucleation diameter of ZnO NWs shows a dependence on the roughness of the Ag thin-film rather than the grain size of the Ag thin-film, as the diameters of 0.5 hour NWs have been seen to increase from 130 nm to 260 nm when the roughness of the Ag thin-film increases from 1.19 nm to 2.60 nm. One possible explanation is that the surface with a larger roughness may have more vacancies that can absorb and receive atoms, affecting the nucleation diameter of ZnO NWs.^36^

As for the ZnO homo seed layer, the ZnO thin-film deposited by e-beam evaporation has (002) grains with relatively high density,^32^ which may be perfectly matched with the basal plane (0001̄) of hydrothermal ZnO nanocrystals. Thus, in our case, the grain size of the seed layer thin-film (44 nm) is similar to the initial diameters of the grown crystals (45 nm), and the density of nucleated grains has been found to be very high. In our opinion, the grain size of the ZnO seed layer plays an important role on the initial diameter of the ZnO NWs. Other researchers have reported similar results of ZnO NWs grown on seed layers prepared by spin-casting ZnO nanoparticles: the grain size of the ZnO homo seed layer has an almost linear relationship with the diameter of ZnO NWs grown after 3 hours,^38^ which indicates the dominant role of the ZnO seed grain size on the initial nucleation diameter of ZnO NWs.

### C. Crystal structure and crystallinity of ZnO NWs

**Fig. 2:**
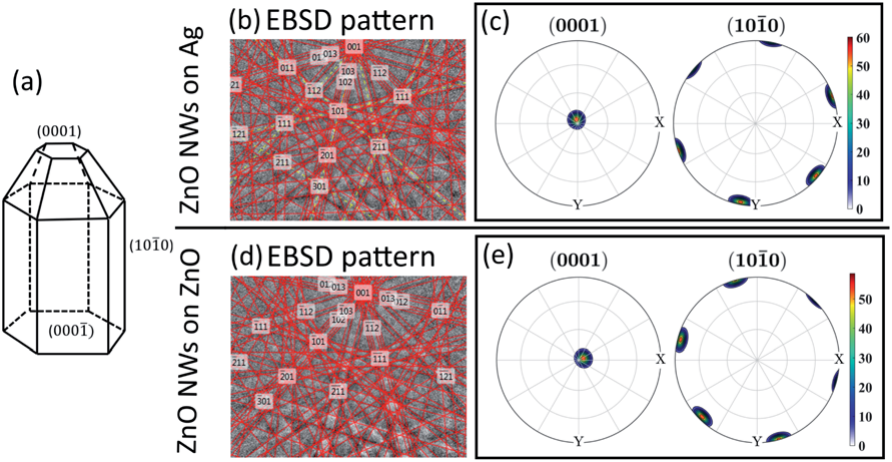
EBSD result of ZnO NWs grown on Ag and ZnO seed layers at 40 mM, 18 hours, and 90 °C. (a) Idealized growth habit of the ZnO crystal (adapted from ref. 39: W. J. Li *et al.*, *J Cryst Growth*, 1999). Electron diffraction pattern of a single point detected on ZnO NWs: (b) ZnO NWs grown on Ag and (d) ZnO NWs grown on ZnO. Pole figures of the (0001) crystal facet and (101̄0) crystal facet, mapped from a single ZnO grain: (c) ZnO NWs grown on Ag and (e) ZnO NWs grown on ZnO.

EBSD has been performed to investigate the crystallinity of ZnO NWs (18 hours, 40 mM) grown on both Ag and ZnO seed layers. The results are presented in Fig. 2.

Fig. 2(a) shows the crystal facets of wurtzite ZnO NWs based on the literature.^39^Fig. 2(b) and (d) show the diffraction pattern formed by our electron beam interacting with a single point on the 70° tilted ZnO NW sample on the Ag and ZnO seed layer, respectively. Assisted by the program AZtecHKL, the diffraction pattern can be fitted with the wurtzite ZnO crystal structure (hexagonal crystal, unit cell *a* = *b* = 3.29 Å, *c* = 5.31 Å).^1^ The successful detection of the electron diffraction pattern and the fitting of crystal atomic parameters in the diffraction pattern indicate that the ZnO NWs exist in the form of wurtzite crystals. In addition, an area of single crystal grains from ZnO NWs grown on both Ag and ZnO seed layers has been mapped, and data from single ZnO grains has been plotted in the pole figure (Fig. 2(c) and (e)) with the assistance of the MTEX program.^31^ It can be seen that for ZnO NWs grown on both Ag and ZnO, the data points (normal direction projected on the unit sphere) of the (0001) crystal facet are all gathered in the centre of the pole figures, and the data points of the (101̄0) crystal facet are gathered on the side of the top-view unit sphere, with a very regular hexagonal shape. The pole figures indicate that ZnO NWs grown on both Ag and ZnO seed layers are vertically oriented single crystals along the [0001] direction.

**Fig. 3:**
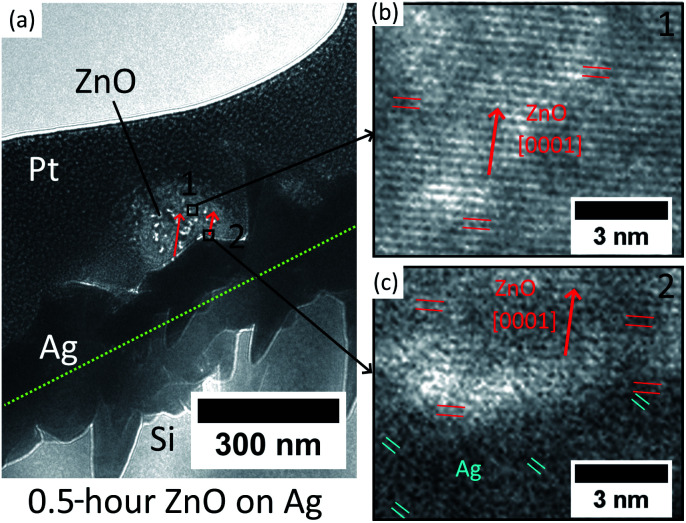
(a) Low-magnification TEM image of ZnO NWs grown on the Ag seed layer at 40 mM, 0.5 hours, and 90 °C. (b) HR-TEM image of the middle of the ZnO nanostructure (position 1). (c) HR-TEM image of the interface between ZnO and Ag (position 2).

In addition, the initial ZnO crystal atomic layers formed on the Ag hetero seed layer have been studied by cross-sectional TEM. The Pt coated ZnO nanostructure with 0.5 hour hydrothermal growth is extracted and thinned by FIB and transferred onto a copper grid. The result is presented in Fig. 3.

The low-magnification TEM image in Fig. 3(a) shows that the Ag thin-film has non-uniform grains on the surface, and the growth direction of ZnO NWs is observed to have a mis-aligned angle to the normal direction of the substrate. For the middle of the ZnO structure (point 1), the HR-TEM image (Fig. 3(b)) confirms that the ZnO is single crystalline and grows epitaxially along the [0001] direction. As for the heterointerface (position 2) shown in Fig. 3(c), [0001] oriented ZnO has been observed to grow epitaxially directly on Ag with a (111) facet. However, as can be seen in Fig. 3(c), the crystal quality of ZnO in the first few nanometres at the heterointerface is observed to be poor, which is probably due to the lattice defects driven by the presence of lattice mismatch and the polycrystalline nature of the Ag thin-film.

The overall HR-TEM results indicate the initial nucleation mechanism of heteroepitaxial growth of ZnO NWs on Ag. The [0001] oriented ZnO crystal has been reported to form preferentially on the [111] oriented Ag grains due to the low lattice mismatch and minimum surface energy.^35,40^ Our e-beam evaporated thin-film Ag possesses a polycrystalline structure, and as can be seen in Fig. 3(a) and (c), Ag grains have relatively random orientations on the surface. At the initial stage of hydrothermal growth, the [0001] oriented ZnO crystals will match with some of the Ag grains with the main orientation of [111] and stochastically nucleated in these positions. The first few atomic layers at the heterointerface may possess a poor crystal quality at the interface due to the presence of low lattice mismatch. The initial growth direction of ZnO NWs is defined by the orientation of (111) Ag facets, and after the nucleation of the initial layers on these (111) Ag facets, ZnO will grow layer-by-layer homogeneously along the [0001] direction with a better crystal quality than the heterointerface.

For the ZnO homo seed layer, some other researchers have obtained the cross-sectional TEM image at the interface between the ZnO seed layer and NWs, and it has been found that ZnO nanowires are grown homoepitaxially on the (002) ZnO seed layer thin-film without clear phase change.^38^ Together with our results on the Ag–ZnO heterointerface, the relationship between seed layers and the as-grown ZnO NWs is clearer. Both Ag and ZnO seed layers can offer nucleation sites, either due to low lattice mismatch facets with [0001] ZnO NWs (Ag seed layer, (111) Ag facets) or due to directly matched ZnO facets with [0001] ZnO NWs (ZnO seed layer, (002) ZnO facets). The crystal quality and the orientation of the seed grains will dominate the initial growth direction of ZnO NWs.

### D. ZnO NWs as a function of different growth times

#### 1. Length and diameter of NWs with different growth times

**Fig. 4:**
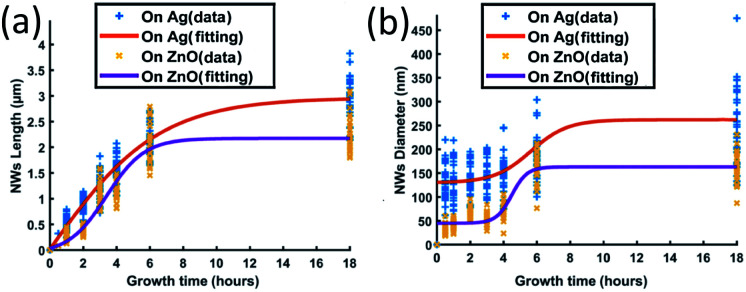
ZnO NWs' (a) length and (b) diameter as a function of growth time; the growth temperature has been fixed at 90 °C and the precursor's concentration has been fixed at 40 mM.

ZnO NW growth as a function of different growth times from 0.5 hours to 18 hours has been studied; the length and the diameter of NWs grown on both Ag and ZnO seed layers have been collected from SEM images and are shown in Fig. 4(a) and (b). During the experiments, the temperature has been fixed at 90 °C, and the precursor concentration has been fixed at 40 mM.

Fig. 4(a) shows that the length of ZnO NWs grown on both Ag and ZnO seed layers starts to increase once the samples are placed in the precursor solution. From the slope of the length curves, it can be observed that the length increases with an overall decreasing growth rate. The growth process is observed to be saturated after a particular time, which is in the range of 8–12 hours for the Ag seed layer and 6 hours for the ZnO seed layer. Fig. 4(b) shows the dependence of the NWs' diameter on growth time for both Ag and ZnO seed layers. During the first few hours, the diameter is found to increase very slowly; then, the diameter can be observed to increase significantly from 3 hours; finally, the diameter is seen to be saturated. The overall trend of NWs' hydrothermal growth on our e-beam evaporated seed layers agrees with the general trend of the NWs grown hydrothermally on the seed layer prepared by spin-casting nanoparticles^24^ and RF sputtering.^41^

Generally, the hydrothermal growth of ZnO NWs includes both physical and chemical processes with complex interactions and uncertainties. The critical difference between NWs grown on hetero and homo seed layers lies in the nucleation of the initial ZnO atomic layers at different interfaces. The ZnO NWs on both Ag and ZnO seed layers are all homo-nucleated once the initial ZnO atomic layer has been formed. After a certain time, thermodynamic equilibrium will be reached when the overall Gibbs free energy remains unchanged; then the ZnO NWs' diameter, length, and density will become saturated.^34,42^ In our hydrothermal growth case, for NWs grown on the Ag seed layer, it takes around 8–12 hours from a 130 nm initial nucleation diameter to reach the saturated state (∼3 μm length, 260 nm diameter), and 6 hours for NWs grown on the ZnO homo seed layer from a 45 nm initial nucleation diameter to achieve the maximum size (∼2.1 μm, 160 nm diameter). ZnO NWs grown on the Ag hetero seed layer are observed to take a longer time to reach the saturated state and have a longer saturated length and larger saturated diameter compared to the NWs grown on the ZnO homo seed layer. The Ag thin film has a rough surface and randomly oriented grains, which may lead to a larger initial diameter on the Ag seed layer as already described. The larger initial diameter of the Ag–ZnO heterointerface may cause a larger saturated diameter when the thermodynamic equilibrium is reached, and it may require a longer time to reach the thermodynamic equilibrium for ZnO NWs grown on the Ag hetero seed layer compared to the ZnO homo seed layer.

#### 2. Density and alignment of NWs with different growth times

**Fig. 5:**
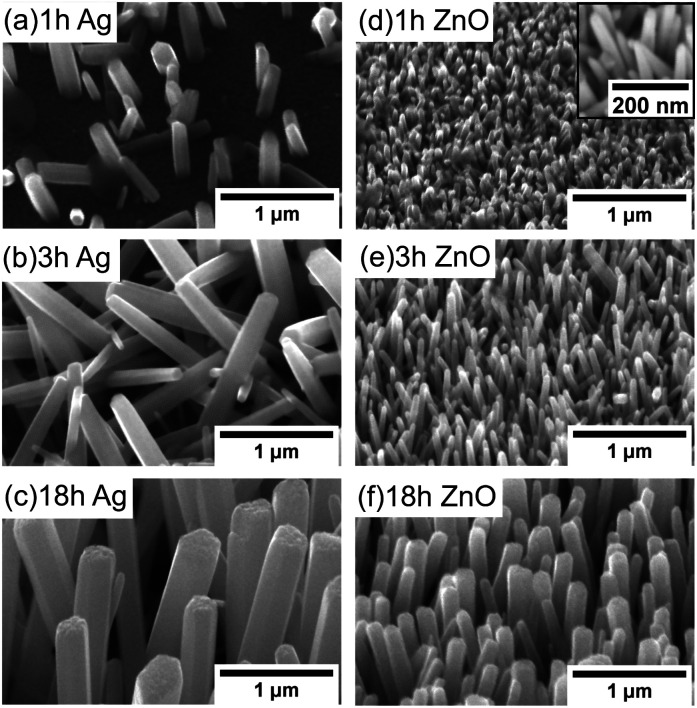
ZnO NWs grown as a function of different times (45° tilted, temperature of 90 °C, precursor concentration of 40 mM), on Ag: (a) 1 hour, (b) 3 hours, and (c) 18 hours; on ZnO: (d) 1 hour with the high magnification image in the inset, (e) 3 hours, and (f) 18 hours.

The density and alignment of NWs with different growth times have been analysed based on the 45° tilted view SEM images shown in Fig. 5. As seen in Fig. 5, the behaviour of ZnO NW growth on Ag hetero and ZnO homo seed layers has been found to be significantly different.

For the Ag seeded ZnO NWs, at 1 hour, only a few NWs with a smaller size grow. Relatively random alignment has been observed (Fig. 5(a)), which is probably due to the random orientation of (111) Ag facets. After around 3 hours, more NWs are observed to be nucleated, and the NWs become longer and start to interact with each other (Fig. 5(b)). When the growth time increases further, although the area coverage of NWs increases, the density in a fixed area decreases due to the increasing diameter of NWs, the limited space and the combined growth of separated NWs, consistent with the results reported by others.^19^ It is worth noting that the NWs' alignment improves significantly when the saturated state has been reached (Fig. 5(c)).

For the ZnO NWs grown on the ZnO homo seed layer, even with 1 hour (Fig. 5(d)), the density of the ZnO NWs is observed to be very high, and the alignment is less random compared to that of the NWs grown on the Ag seed layer. One possible reason is that every point in the ZnO seed layer is equally suitable for nucleation, and hence the alignment mainly depends on the surface properties.^43^ When the growth time increases further, the alignment of the ZnO NWs also improves, similar to the NWs grown on the Ag seed layer. Overall, based on our observation, the alignment of NWs shows a dependence on the density of NWs and the interaction between neighbouring NWs, while the gravity gradient,^44^ the presence of lattice mismatch between NWs and hetero seed layers,^35^ and the surface morphology of the seed layer^45^ have been used to explain the degree of alignment of ZnO NWs.

### E. The influence of different precursor concentrations

**Fig. 6:**
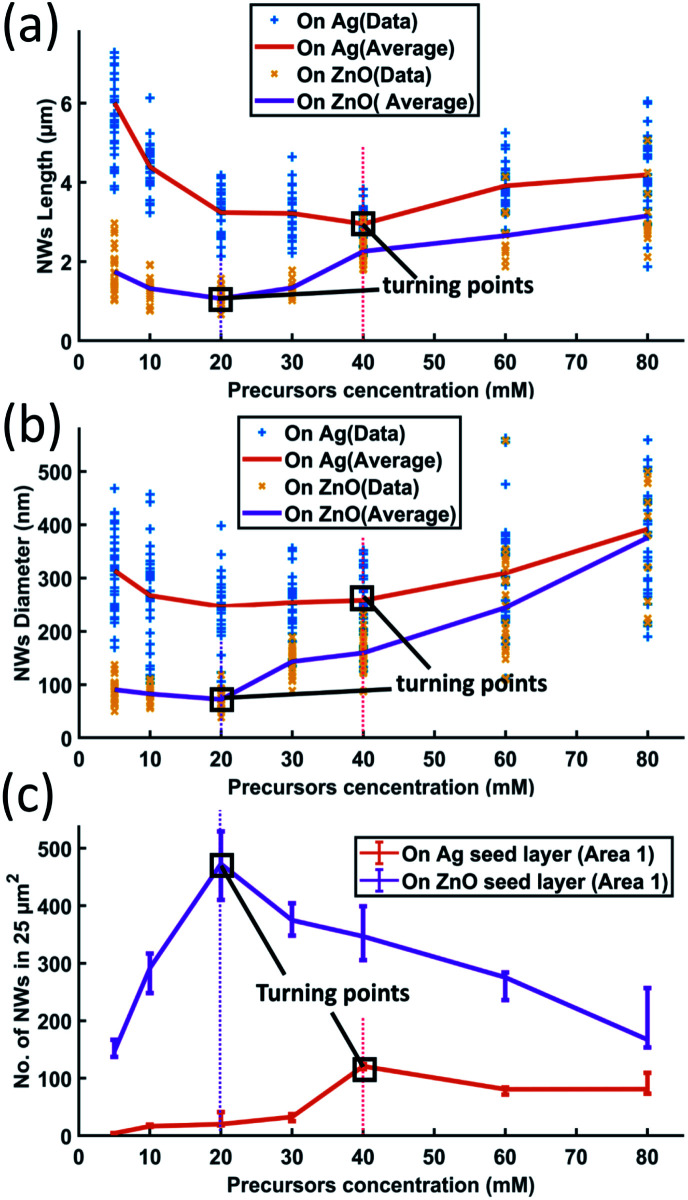
ZnO NW growth as a function of different precursor concentrations (18 hours growth at a temperature of 90 °C): (a) length; (b) diameter; and (c) density.

**Fig. 7:**
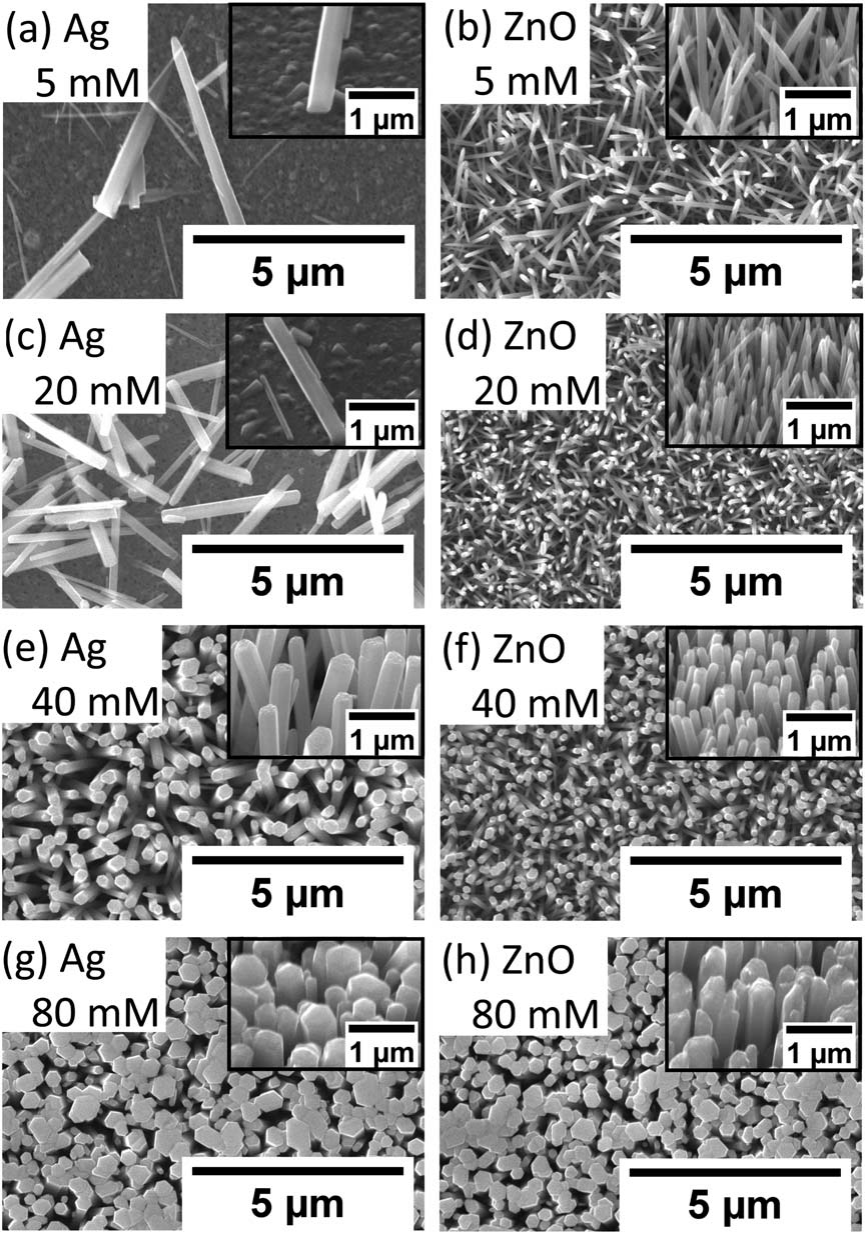
ZnO NW growth (90 °C, 18 hours) on Ag and ZnO seed layers as a function of different precursor concentrations (top view and tilted 45°). On the Ag seed layer: (a) 5 mM, (c) 20 mM, (e) 40 mM, and (g) 80 mM; on the ZnO seed layer: ZnO (b) 5 mM, (d) 20 mM, (f) 40 mM, and (h) 80 mM.

To investigate the influence of the precursor concentration on the growth of ZnO NWs of Ag and ZnO seed layers, equimolar concentrations of zinc nitrate hexahydrate and HMTA have been changed from 5 mM to 80 mM. The growth time has been fixed at 18 hours, which can be treated as the saturated state for 40 mM cases. Fig. 6(a)–(c) show the length, diameter, and density of ZnO NWs grown on both Ag and ZnO seed layers as a function of precursor concentration. The detailed SEM images of NWs are shown in Fig. 7.

It can be seen in Fig. 6 and 7 that, overall, the NWs grown on the Ag seed layer have a longer average length and larger average diameter than those on the ZnO seed layer, but the density of NWs on the Ag seed layer is significantly lower than that of NWs on the ZnO seed layer. However, for NWs grown on both Ag and ZnO seed layers as a function of the precursor concentration, the length and diameter of NWs first decrease and then increase; on the other hand, the density increases first and decreases as the precursor concentration increases. With the precursor concentrations being varied from 5 mM to 80 mM, three different cases have been observed:

(1) At very low precursor concentrations (5 mM–10 mM), very long, medium diameter NWs can be seen (Fig. 7(a) and (b)). This is probably because the (002) facet of ZnO NWs has a minimum facet energy,^35^ which is observed to be the main growth direction when chemical reagents are limited, and the overall density of NWs is observed to be low.

(2) As the precursor concentration increases, more nucleation points can be initiated by more chemical reactions. Then, the overall density of ZnO NWs is observed to increase very fast. However, theoretically, there could be chemical competition between different NWs, which may cause the NWs' average length and diameter to be limited.^46^ Based on this hypothesis, both on Ag and ZnO thin films, there will be a turning point with a minimum length but maximum density, which can be seen in Fig. 6(c). For the Ag seed layer, the point is observed to be 40 mM (Fig. 7(e)), while it is around 20 mM for the ZnO seed layer (Fig. 7(c)).

(3) When the precursor concentration increases further (around 40 mM–80 mM), the chemical reagents could be assumed to be enough for every single NW, and the length and diameter are observed to grow further. However, space and mass transport can still limit the crystal growth.^47^ Some NWs are observed to combine and form a bigger one, which can lead to a decreasing trend of NW density, which can be observed in Fig. 7(f) and (h). It can be predicted that when the precursor concentration is very high, a relatively uniform ZnO thin film can be formed, which has been mentioned in the case of other seed layers.^48^

**Fig. 8:**
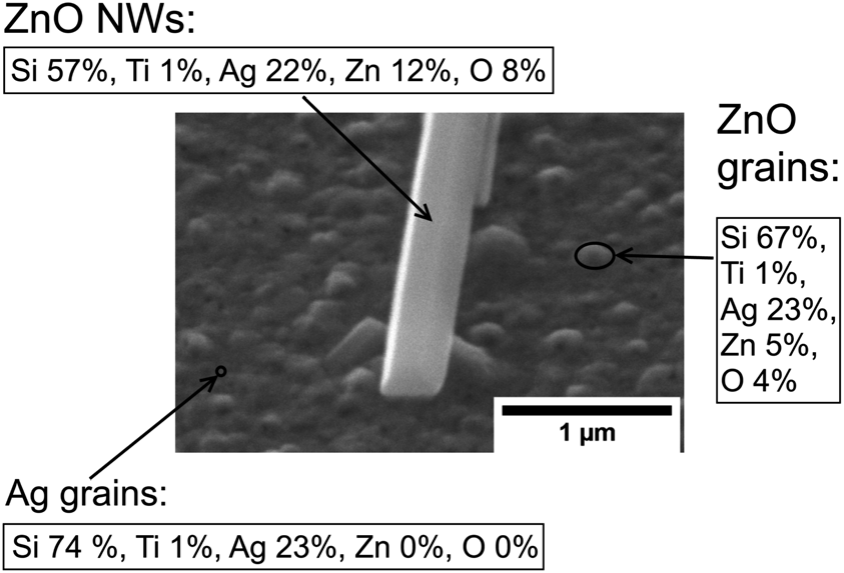
SEM image of ZnO NWs grown on Ag (45 °C tilted) at a precursor concentration of 5 mM and growth time of 18 hours. EDX data (elemental percentage) have been shown at the points of ZnO NWs, ZnO grains, and Ag grains. The electron beam penetration depth is ∼1 μm; carbon and other contaminants have been ignored.

It is worth noting that a different growth behaviour between ZnO NWs grown on Ag and ZnO seed layers has been observed at low precursor concentrations (Fig. 7(a)–(d)). At precursor concentrations below 20 mM, the ZnO NWs grown on the Ag hetero seed layer have been found to exhibit random growth directions and lower density compared to ZnO NWs grown on the ZnO homo seed layer. To investigate the reason for the disordered growth in the hetero growth case (ZnO NWs/Ag), EDX has been used to analyse the ZnO NWs grown on the Ag seed layer (5 mM, 18 hours), which are shown in Fig. 8.

As can be seen in Fig. 8, in addition to the larger ZnO NWs as already described, smaller and denser grains have been observed on the Ag surface. EDX data show that the small grains formed on the surface are detected to be ZnO instead of Ag. The diameter of the ZnO grains is around half that of the larger ZnO NWs, and the height of grains is significantly lower than the NWs' length. The existence of ZnO grains at low precursor concentrations and long growth times suggested that NWs' growth may be driven by Ostwald ripening.^49^ At a low precursor concentration, relatively high-density grains will overcome the hetero energy barrier to form on the Ag surface. However, statistically, only a few ZnO grains can absorb more precursors as they become the dominant NWs in this limited precursor concentration regime. The formation of new grains and the mass transport of precursors to other existing small grains will be limited. Thus, the overall density of ZnO NWs grown on Ag will be low under limited chemical concentrations. In addition, the influence of neighbouring NWs on the alignment of ZnO NWs will be negligible, and the growth direction of ZnO NWs will be dominated by the randomly oriented (111) Ag facets. All the effects described above may contribute to the explanation of the disordered growth of ZnO NWs at low precursor concentrations.

### F. Optical properties: deep level emission defect study

#### 1. PL spectra of Ag and ZnO seeded ZnO NWs as a function of growth time

**Fig. 9:**
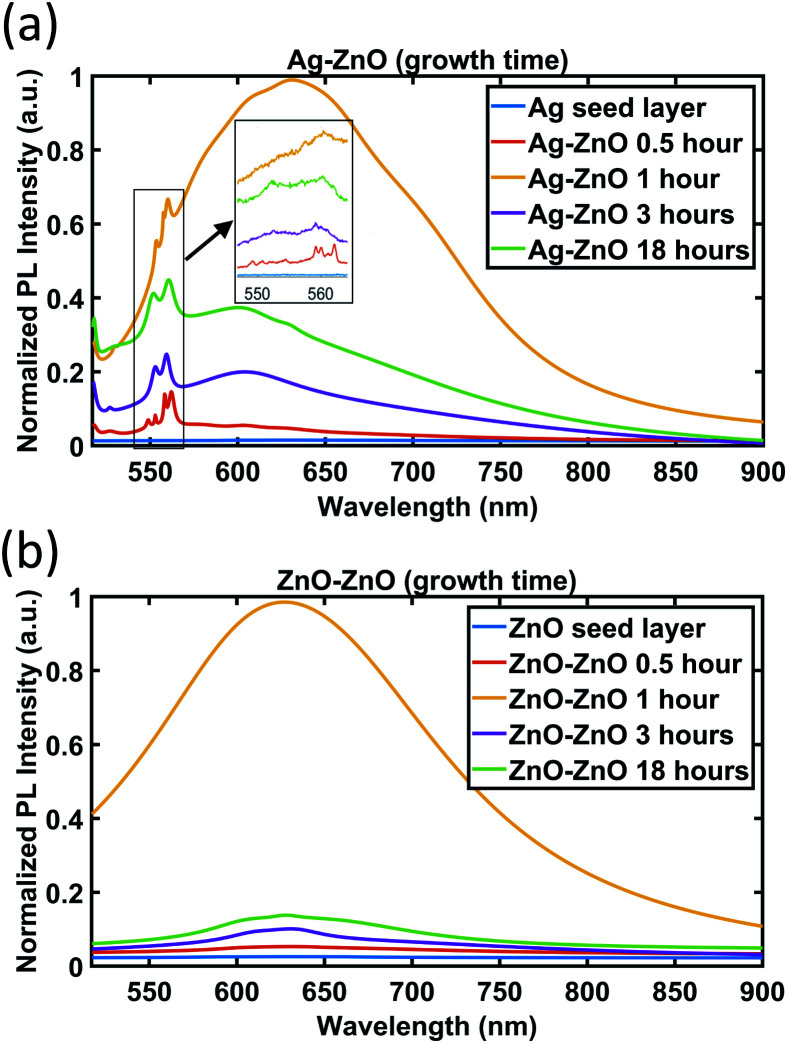
PL spectra of ZnO NWs grown on (a) Ag and (b) ZnO seed layers as a function of growth time. For the Ag seed layer, the zoomed-in spectra between 550 and 560 nm are shown in the inset.

As a wide bandgap semiconductor, ZnO materials are reported to exhibit a band emission peak around 380 nm in the PL spectra, which is in the near UV (ultraviolet) range.^1,35,50^ However, for ZnO NWs, there are many deep level emission peaks in the visible range due to either internal vacancies or external defects especially when the heterointerface exists.^50,51^ In the past few years, deep level emission peaks of the ZnO material and related heterostructures in the visible range have drawn a lot of interest for both defect mechanism study and in optical applications such as a new candidate for visible LEDs (light-emitting diodes).^50,52,53^ In our work, room-temperature PL spectra in the visible range of 517 nm to 900 nm for our ZnO NWs grown on both Ag and ZnO seed layers have been studied. The PL spectra in the 517 nm to 900 nm range have been obtained from the ZnO NWs as a function of growth time and are presented in Fig. 9.

As can be seen in Fig. 9, no apparent peak has been observed in both Ag and ZnO seed layers without ZnO NWs. For ZnO NWs grown on both Ag and ZnO seed layers, broad peaks between 600 nm and 650 nm have been observed. In the literature, many researchers have reported this orange/red emission broad peak, which is attributed to the deep level emission defects induced by oxygen vacancies or interstitials.^54,55^ However, the exact origin (either vacancies or interstitials) of these broad peaks is still controversial.^50^ Together with the broad peak, the ZnO NWs grown on the Ag seed layer have been observed to exhibit a few dominant peaks at around 550 nm to 560 nm. To our knowledge, these green emission peaks at ∼550 nm of ZnO NWs grown on the e-beam evaporated Ag seed layer have not been reported before. Instead of highly intensive narrow green peaks, there are many reports on broad green peaks from ZnO materials in the literature,^56,57^ also attributed to oxygen-related defects. In our case, it is believed that the narrow peaks in the range 550 nm to 560 nm are possibly related to lattice defects initiated from the heterointerface between the e-beam Ag seed layer and ZnO NWs.

In Fig. 9, the broad peaks (600 nm–650 nm) behave similarly on both Ag and ZnO seeded NWs as a function of time. The intensity of the broad peak has been observed to be the highest for the 1 hour growth cases, which suggests that oxygen-related defects in ZnO NWs become dominant in the first 1 hour of growth. From 1 hour to 3 hours, the intensity of the broad peak drops dramatically. In the period from 3 hours to 18 hours, the intensity of the broad peak increases again slightly for the 3 hour case.

The evolution of the green emission peaks at around 550 nm to 560 nm, for ZnO NWs grown on Ag seed layers, is presented in the inset of Fig. 9(a). It can be seen that around 4 to 5 peaks appear in the range of 550 nm to 560 nm at a 0.5 hour growth time, which may be related to the defects during the formation of the initial heterointerface between Ag and ZnO. This result agrees with the HR-TEM result (Fig. 3(c)) that at the interface between 0.5 hour grown ZnO on Ag thin films, the crystal quality is poor due to the presence of defects. As the growth time increases and the ZnO NWs grow denser and larger, these peaks become stable and combine into two major peaks, at 553 nm and 559 nm. The 553 nm peak is significantly less intense than the 559 nm peak before 3 hours. However, at 18 hours, the intensity of the 553 nm peak increases and becomes similar to the 559 nm peak's intensity. These observations might indicate that the type of defects in ZnO nanowires becomes more stable when growth time increases.

#### 2. PL spectra of Ag and ZnO seeded ZnO NWs as a function of precursor concentration

**Fig. 10:**
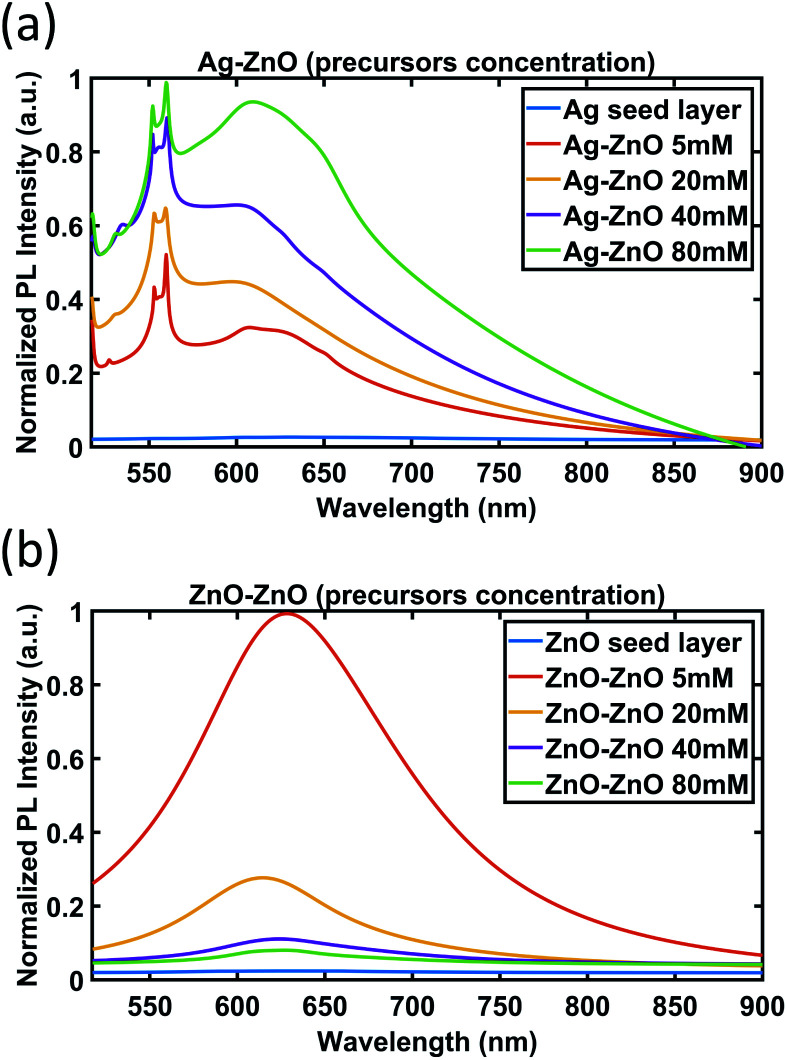
PL spectra of ZnO NWs grown on (a) Ag and (b) ZnO seed layers as a function of precursor concentration (growth time of 18 hours).

To investigate the optical properties of ZnO NWs grown on Ag and ZnO seed layers further, the PL spectra have been obtained for the ZnO NWs grown with different precursor concentrations (growth time of 18 hours) and are shown in Fig. 10.

As can be seen in Fig. 10(a), for ZnO NWs grown on the Ag seed layer, as the precursor concentration increases, the intensity of the peaks (553 nm and 559 nm) and the broad peaks (from 600 nm to 650 nm) has been observed to have an increasing trend. However, in Fig. 10(b), for ZnO NWs grown on the ZnO seed layer, as the precursor concentration increases, the intensity of broad peaks has been observed to show a decreasing trend.

The completely different trends of peak intensity for ZnO NWs grown on Ag and ZnO seed layers as a function of precursor concentration are indicative of the different role of defects in the photoluminescence of the ZnO NWs. The dominant defects in ZnO NWs grown on the Ag seed layer are believed to be lattice defects at the heterointerface between Ag and ZnO NWs, which are attributed to the peaks observed between 553 nm and 559 nm. As more precursors are supplied, more ZnO NWs will nucleate on the Ag surface, and the level of defects will increase; hence the PL intensity related to the defect peaks increases.

However, for ZnO NWs grown on the ZnO seed layer, the density of ZnO NWs has been observed to be high even with low precursor concentrations. The dominant defects in ZnO NWs grown on the ZnO seed layer are believed to be oxygen-related defects of ZnO NWs, which are related to the broad peaks ranging from 600 nm to 650 nm. The broad peaks have been observed to show a decreasing trend as the precursor concentration increases. This observation is suggestive of the presence of fewer oxygen-related defects, and hence higher quality ZnO seeded ZnO NWs as the precursor concentration increases. A possible explanation may be the fact that the oxygen-related defects are associated more with the degree of exposed outer surfaces of NWs. It can be seen that as the precursor concentration increases, the diameters of the ZnO seeded ZnO NWs grown increase and the density of the NWs decreases (see Fig. 6(b) and (c) and Fig. 7), hence exposing fewer NW surfaces. On the other hand, for Ag seeded ZnO NWs, the oxygen-related defect peaks (from 600 nm to 650 nm) have been observed to increase as the precursor concentration increases. It can be seen from Fig. 6(c) and 7 that the amount of exposed outer surfaces of Ag seeded ZnO NWs increases as the precursor concentration increases. Overall, these observations suggest that there is a certain influence of the amount of exposed surface area of NWs on the intensity of oxygen-related PL broad peaks.

## IV. Conclusions

The surface properties of e-beam evaporated Ag and ZnO seed layers, the ZnO–Ag and ZnO–ZnO nucleation interfaces, the crystallinity of ZnO NWs, and the growth mechanism of ZnO NWs on e-beam evaporated Ag and ZnO seed layers have been investigated. E-beam evaporation deposited Ag and ZnO seed layers show an average roughness of less than 2.6 nm and a grain size from 28–44 nm. It has been found that the roughness of the Ag seed layer and the grain size of the ZnO seed layers play more dominant roles in affecting the initial diameter of the ZnO NWs. The initial diameters of the ZnO NWs on the Ag seed layers (∼130 nm) have been found to be 4 to 6 times larger than the grain size of the Ag seed layers while ZnO NWs grown on ZnO seed layers (45 nm) have similar initial diameters. ZnO NWs grown on both Ag and ZnO seed layers show [0001] oriented wurtzite crystal structures based on both EBSD results and HR-TEM results. In addition, from the HR-TEM results, the crystal quality of ZnO NWs at the initial heterointerface has been observed to be poorer than that in the middle part of ZnO NWs. From the investigation of the ZnO NWs grown on Ag and ZnO seed layers as a function of growth time, it has been found that ZnO NWs on Ag have a longer length, larger diameter, and less density and are more randomly aligned than the ZnO NWs on the ZnO seed layer. As for the dependence of length, diameter and density of the ZnO NWs on the precursor concentration, it has been found that there is a turning point with a minimum length and maximum density for both types of ZnO NWs; on the Ag seed layer, the turning point is observed to be at 40 mM and for the ZnO seed layer, the turning point is at 20 mM. Moreover, both the Ag–ZnO NW heterostructure and ZnO–ZnO NW homostructure show a broad PL peak at 600 nm to 650 nm wavelengths; in particular, for the Ag–ZnO NW heterostructure, additional green emission peaks at around 553 nm and 559 nm have been observed. Furthermore, as the precursor concentration increases, the defect-related PL peaks have been observed to increase for the Ag–ZnO NW heterostructure while the defect-related PL peaks decrease for the ZnO–ZnO NW homostructure. The outcome of this study can be helpful in understanding the mechanism of ZnO homo and hetero growth and provides a guideline for the microfabrication of future ZnO-based devices such as piezoelectric force micro-sensors, nano-generators, and optoelectronic devices.

## Conflicts of interest

There are no conflicts to declare.

## Supplementary Material

NA-002-C9NA00553F-s001
