# Supplementary material for: Comparison of ZnO nanowires grown on e-beam evaporated Ag and ZnO seed layers
Source: Nanoscale Adv. 2020 May 19;2(7):2814–23. doi: 10.1039/c9na00553f (PMC9418495; doi:10.1039/c9na00553f)
Supplement: NA-002-C9NA00553F-s001 [file NA-002-C9NA00553F-s001.pdf]

Electronic Supplementary Material (ESI) for Nanoscale Advances.
This journal is © The Royal Society of Chemistry 2020

Electronic supplementary information for

Comparison of ZnO Nanowires Grown on E-beam Evaporated Ag and ZnO seed layers

Yulin Geng*[a], Karina Jeronimo, Muhammad Ammar Bin Che Mahzan, Peter Lomax, Enrico Mastropaolo, Rebecca Cheung

[a.] University of Edinburgh, Institute for Integrated Micro and Nano Systems, Scottish Microelectronics Centre, Alexander Crum Brown Road Edinburgh EH9 3FF, Scotland, United Kingdom.

Email address correspondence to: yulin.geng@ed.ac.uk

TEM sample preparation: FIB

The cross-sectional TEM sample (ZnO NWs grown on Ag seed layer) has been prepared by the focus ion-beam (FIB). Fig.S1 shows the SEM image taken during the FIB preparation of TEM sample.

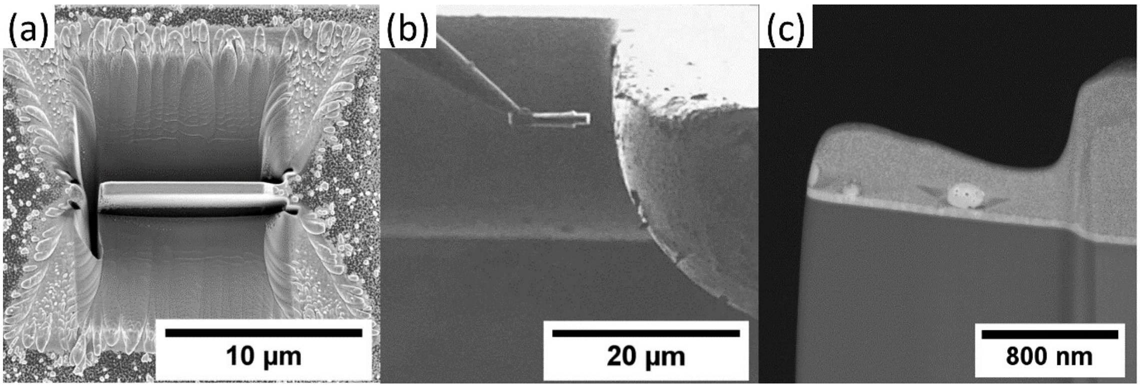

Fig.S1. The SEM image during the FIB preparation of TEM sample. (a). The selected area of the sample is coated with platinum, and the surrounding and the bottom of the selective area are etched with ion-beam. (b). The transfer of the sample onto the copper grid using a probe. (c). Sample thinning by ion-beam (cross-sectional view).

Firstly, an area (9 μm× 0.5 μm) with ZnO NWs grown on Ag is selected. Platinum (Pt) is deposited to coat the area and protect the area from the high energy ion-beam. Then, high energy ion-beam is used to etch the surrounding and bottom of the selected area (Fig.S1(a)). A probe is fused to the top Pt of the selected area by ion-beam, followed by removing the supporting side of the selected area from the substrate. At this stage, the selected area has been fully extracted from the substrate.

The next stage is to transfer the selected area to the copper grid. The probe carries the selected area to one side of the copper grid (Fig.S1(b). Once the select area touchs the side of the copper, the focused ion-beam is used to fuse the selected area into the copper and the connection of the probe to the selected area is then removed.

The last stage of the sample preparation is to thin the sample for TEM. The sample is thinned from the side (Fig.S1(c)) very carefully by the focused ion-beam. The resulting thickness of the sample achieved is around 50 nm.

After the sample preparation, the cross-sectional selected area TEM is performed by FEI F20 Cryo TEM at 200 kV. The result images have been shown in the main content of the manuscript (Fig.3).
